# Case Report: Pyrrolizidine alkaloid-induced hepatic sinusoidal obstruction syndrome misdiagnosed as ovarian malignancy

**DOI:** 10.3389/fphar.2026.1833645

**Published:** 2026-09-30

**Authors:** Ke Wang, Shuhua Wang, Yijuan Wang, Jing Lu, Huiling Wang, Fangfang Hua, Jun Yang

**Affiliations:** Department of Gynecology, The first Affiliate Hospital of Henan Medical University, Xinxiang, China

**Keywords:** ascites, Gynura segetum (Tusanqi), hepatic sinusoidal obstruction syndrome (HSOS), misdiagnosis, ovarian cancer, pyrrolizidine alkaloids (PAs)

## Abstract

**Background:**

Hepatic Sinusoidal Obstruction Syndrome (HSOS), formerly known as Hepatic Veno-Occlusive Disease (HVOD), is a rare and life-threatening liver condition. It is often caused by the ingestion of pyrrolizidine alkaloids (PAs), which are found in plants such as Gynura segetum (Tusanqi). Because its clinical presentation is non-specific, HSOS is frequently misdiagnosed.

**Case Presentation:**

A 72-year-old female with a 5-year history of type 2 diabetes presented with abdominal distension and anorexia for 2 weeks. Initial examinations revealed massive ascites and significantly elevated tumor markers (Cancer Antigen 125(CA125): 195.0 U/mL, Human Epididymis Protein 4(HE4): 120.1 pmol/L). Imaging suggested potential ovarian cancer, leading to diagnostic laparoscopy and bilateral adnexectomy. However, intraoperative findings and post-operative pathology showed no evidence of malignancy, identifying only a serous cystadenoma. Subsequent detailed history-taking revealed long-term ingestion of “Tusanqi.” Re-evaluation *via* contrast-enhanced CT and MRCP showed characteristic patterns: patchy enhancement of hepatic parenchyma (“geographic” pattern), narrow hepatic veins, and signs of portal hypertension. The patient was finally diagnosed with PA-induced HSOS. Following a regimen of anticoagulation (low-molecular-weight heparin), liver protection, and diuretics, the patient’s abdominal distension relieved, and liver function markers showed partial recovery. The patient underwent laparoscopic bilateral adnexectomy due to a high suspicion of ovarian cancer, but the postoperative pathology ruled out malignant tumors, resulting in unnecessary surgical trauma. This article aims to improve the warning effect.

**Conclusion:**

This case highlights the clinical challenge of distinguishing HSOS from abdominal malignancies. A thorough dietary/herbal history and characteristic “geographic” enhancement on imaging are pivotal for avoiding unnecessary surgical interventions.

## 1 Introduction

Ovarian malignancies are primary differential diagnoses for malignant ascites due to their insidious onset and rapid progression. However, certain types of toxic liver injury can strikingly mimic the clinical, biochemical, and even radiological features of peritoneal carcinomatosis, frequently leading to diagnostic pitfalls (Crawford, 2010; Yang et al., 2019). Hepatic Sinusoidal Obstruction Syndrome (HSOS), formerly known as Hepatic Veno-Occlusive Disease (HVOD), is a clinical syndrome characterized by jaundice, hepatomegaly, ascites, and portal hypertension resulting from the obstruction of hepatic venous outflow due to sinusoidal endothelial cell damage (Crawford, 2010; Valla, 2003).

In China, the predominant etiology of HSOS is the accidental or medicinal ingestion of plants containing pyrrolizidine alkaloids (PAs), most notably Gynura segetum, commonly referred to as “Tusanqi” (Yang et al., 2019; Lin et al., 2011). The toxic metabolites produced during the hepatic metabolism of PAs cause swelling and necrosis of sinusoidal endothelial cells, eventually leading to the narrowing or occlusion of intrahepatic venules (Zuckerman et al., 1999; Angelico, 2024; Northup et al., 2021). Because early-stage HSOS can present with significant elevations in tumor markers (e.g., CA125) (Zuckerman et al., 1999; Topalak et al., 2002), massive ascites, and pseudo-tumoral patterns on imaging, it is frequently misidentified as advanced abdominal malignancy or peritoneal metastasis. Such misdiagnosis often leads to unnecessary surgical interventions (e.g., exploratory laparotomy or adnexectomy) and delayed initiation of appropriate anticoagulation and supportive therapies (Dai et al., 2007; Huang et al., 2023).

This report describes a case of a 72-year-old female patient who was highly suspected of having ovarian cancer based on massive ascites, elevated CA125, and suspicious imaging findings. After undergoing surgical adnexectomy, a thorough review of her dietary history and re-evaluation of characteristic imaging patterns confirmed a diagnosis of hepatic sinusoidal obstruction syndrome (HSOS) induced by “Tusanqi” poisoning. This case highlights the importance for clinicians to remain vigilant about plant-derived drug-induced liver injury when encountering unexplained ascites and elevated tumor markers. A detailed history of herbal medicine use remains essential to avoid unnecessary medical intervention (Zhang et al., 2025).

## 2 Case treatment progress

### 2.1 Initial presentation and preliminary investigation

The patient, a 72-year-old female, was admitted with a 5-year history of diabetes and a 15-day history of abdominal distension and anorexia. The initial diagnoses were type 2 diabetes mellitus and ascites of unknown origin. Initial findings included an HbA1c level of 8.8% and significant ascites confirmed by abdominal ultrasound. Tumor marker testing revealed elevated levels of CA125:195.0 U/mL, CA199:114.1 U/mL, and HE4 120.1 pmol/L, raising suspicion of ovarian malignancy. Pelvic MRI suggested potential ovarian cancer with massive ascites and peritoneal thickening (Figure 1A) (CA125: Normal reference range is < 35 U/mL.CA19-9: Normal reference range is <37 U/mL.HE4: Normal reference values vary by menopausal status <140 pmol/L for premenopausal women and <92 pmol/L for postmenopausal women. The above reference values are for general guidance only; please refer to the reference range provided by the testing laboratory for clinical interpretation.)

**FIGURE 1:**
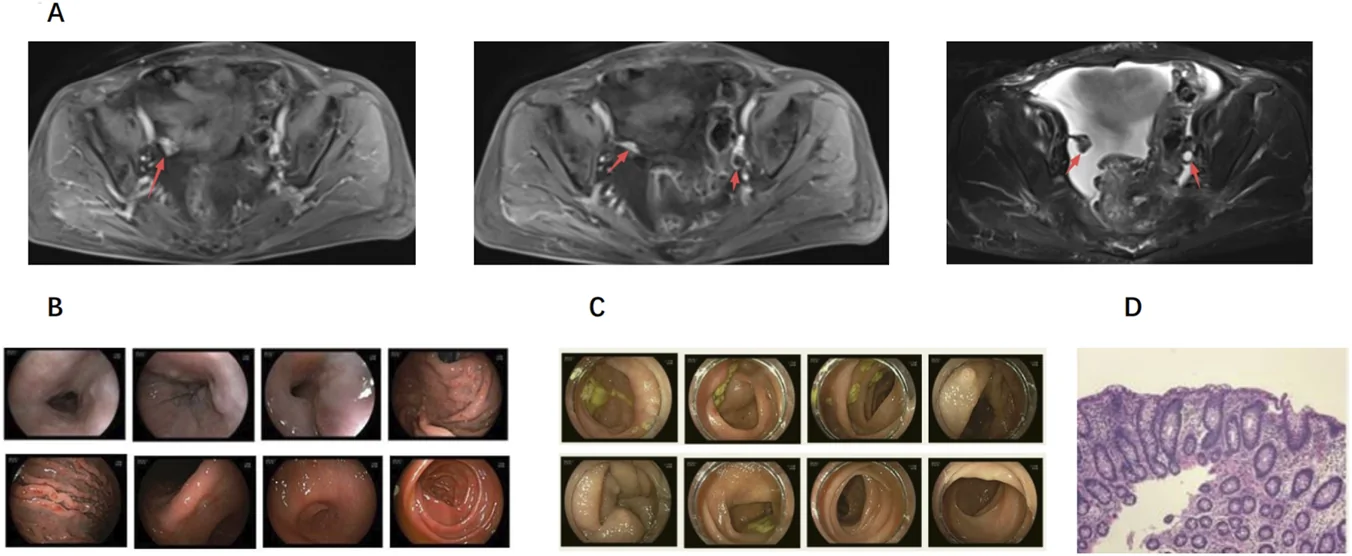
Initial presentation and preliminary investigation. **(A)** MRI revealed increased soft tissue shadows in the bilateral adnexal regions, suspicious for ovarian cancer, accompanied by massive abdominopelvic effusion. **(B)** Gastroscopy revealed mild esophageal varices, esophagitis, and chronic active gastritis. **(C)** Colonoscopy revealed multiple colonic polyps and melanosis col. **(D)** Pathological biopsy of the transverse colon revealed an inflammatory polyp.

Gastroscopy revealed mild esophageal varices and chronic active gastritis (Figure 1B), while colonoscopy showed multiple polyps and melanosis coli (Figures 1C,D). The purpose and significance of gastrointestinal endoscopy in this patient is to exclude metastatic cancer from the digestive tract. The ovary is a common metastatic site of intra-abdominal malignant tumors. Gastric cancer, colorectal cancer, pancreatic cancer and other gastrointestinal tumors often metastasize to the ovary to form “Krukenberg tumor”. The clinical manifestations of Krukenberg tumor (pelvic mass, ascites, CA125 elevation) are very similar to primary ovarian cancer. Gastrointestinal endoscopy is helpful to find the primary gastrointestinal lesions and determine the origin of the tumor.

Diagnostic peritoneal puncture was performed on admission, and ascites cytology results: lymphocytes, macrophages, mesothelial cells and degenerative cells were found in ascites. Ascites albumin: 13.8 g/L, adenosine dehydrogenase (ADA):3.1U/L, lactate dehydrogenase (LDH): 65U/L ascites related examination results are found in Tables 1, 2.

**TABLE 1:** Ascites was routinely examined.

Abbreviation	Item	Result	Unit	Reference range
YS	Color	Yellow	​	Colorless
JYWG	Turbidity	Clear	​	Clear
SFNJ	Agglutination	None	​	None
NDBSY	Rivalta test	Positive (1+)	+	Negative
YHXBS	Nucleated cell count	130	×10^6^/L	​
DYHJS	Polymorphonuclear cell percentage	20	%	​
DGHJSHU	Mononuclear cell percentage	80	%	​
JPXB	Mesothelial cell percentage	-	%	​
JJQT	Microscopic examination - others	-	​	​

**TABLE 2:** Relevant laboratory tests after admission.

Relevant laboratory tests after admission	At admission (pre-operation)	After treatment	Follow-up period
Aspartate aminotransferase (AST)	64 U/L	33 U/L	29 U/L
Alanine aminotransferase (ALT)	41 U/L	17 U/L	16 U/L
Alkaline phosphatase (ALP)	265 U/L	145 U/L	120 U/L
Gamma-glutamyl transferase (GGT)	454 U/L	161 U/L	186 U/L
Total bilirubin	15.6 µmol/L	24.8 µmol/L	28.3 µmol/L
Direct bilirubin	6.4 µmol/L	13 µmol/L	17.1 µmol/L
Albumin	31.3 g/L	34.5 g/L	30.7 g/L
International normalized ratio (INR)	0.87	0.82	0.84
Platelet count	153 × 10^9^/L	92 × 10^9^/L	130 × 10^9^/L
Serum creatinine	72.1 μmol/L	61.3 μmol/L	81.3 μmol/L

### 2.2 Surgical exploration and pathological diagnosis

Given the constellation of postmenopausal status, rapid accumulation of massive ascites, markedly elevated ovarian tumor markers (CA125: 195.0 U/mL, HE4: 120.1 pmol/L, CA19-9: 114.1 U/mL), and pelvic MRI findings suggestive of bilateral adnexal soft-tissue lesions with peritoneal thickening, an advanced ovarian malignancy (e.g., epithelial ovarian carcinoma with peritoneal carcinomatosis) could not be excluded. Following extensive multidisciplinary discussion and thorough informed consent with the patient and her family, a diagnostic laparoscopy with prospective surgical staging was indicated. The limitation of this case study is that the operation was performed without completing liver specialist consultation and collecting the history of taking Chinese herbal medicine in detail. The procedure was performed on 12 August 2025. Intraoperative findings: approximately 1,000 mL of straw-colored fluid was aspirated. Inspection of the uterus, ovaries, liver, spleen, and gastrointestinal tract revealed no gross evidence of tumor or metastasis.

Procedures: A laparoscopic bilateral adnexectomy and multi-point peritoneal biopsies were performed. Pathology: Frozen sections and subsequent paraffin pathology identified a “serous cystadenoma of the left ovary,” with no evidence of malignancy in the adnexa or peritoneum Ascites cytopathological diagnosis: lymphocytes, macrophages and mesothelial cells were found. (Figures 2A–C).

**FIGURE 2:**
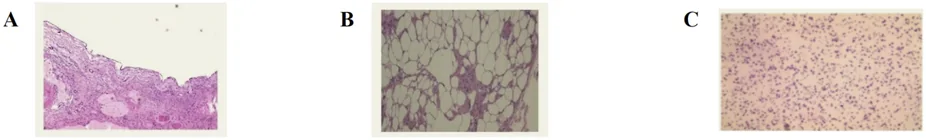
Surgical exploration and pathological diagnosis. **(A)** Pathology results revealed a left adnexal serous cystadenoma, with no definite evidence of malignancy. **(B)** Pathology of the intraoperative peritoneal biopsy revealed fibrocollagenous and adipose tissue, with no tumor components identified. **(C)** Pathological diagnosis of ascites cells: lymphocytes, macrophages and mesothelial cells were found.

### 2.3 Multidisciplinary consultation and definitive diagnosis

Because the cause of ascites remained unclear after surgery, consultations were sought with the Tuberculosis and Infectious Disease departments. Final diagnosis: Based on the herbal history and imaging findings, the patient was diagnosed with hepatic sinusoidal obstruction syndrome (HSOS) induced by pyrrolizidine alkaloids (Tusanqi poisoning).

#### 2.3.1 Diagnostic criteria for HSOS

The “Nanjing Criteria” for hepatic sinusoidal obstruction syndrome (HSOS) were proposed in the 2017 Expert Consensus on the Diagnosis and Treatment of Pyrrole Alkaloid-Related Hepatic Sinusoidal Obstruction Syndrome. These criteria are specific for the clinical diagnosis of HSOS caused by pyrrole alkaloids (PA). The core requirements include a clear history of PA intake, meeting three clinical or auxiliary conditions (or a pathological diagnosis), and excluding other liver diseases. The diagnostic criteria are as follows (if one is met): 1. Clinical diagnosis: all three of the following are present: (1) a clear history of taking PA-containing plants (e.g., Gynura segetum, also known as senli-kuang); (2) typical clinical manifestations: abdominal distension and/or pain in the liver region, hepatomegaly, and ascites; (3) abnormalities on auxiliary examination: elevated serum total bilirubin or other abnormal liver function, and typical enhanced CT/MRI findings (map-like or mottled heterogeneous enhancement, no development or stenosis of hepatic veins, *etc.*). 2. Pathological diagnosis: a clear history of PA intake plus liver biopsy showing hepatic sinusoidal endothelial injury, shedding, and sinus dilatation in area I of the hepatic acinus. Congestion and venular occlusion were excluded, and other liver diseases were excluded.

#### 2.3.2 This case is based on the diagnosis

1. The patient’s medical history was reviewed, and he had been receiving long-term oral Chinese herbal medicine containing pyrrole alkaloids (Gynura segetum, Tusanqi). 2. The clinical manifestations were massive ascites and abdominal distension in a short period of time. 3. Auxiliary examination results: abnormal liver function: total protein:60.5 g/L↓, albumin:31.3 g/L↓, glutamyl transpeptidase:454U/L↑, bile acid:28.3umol/L↑.Comprehensive etiologic screening for viral hepatitis (HAV, HBV, HCV, HEV), autoimmune liver diseases (ANA, ASMA, AMA-M2, IgG4), and systemic vasculitis were strictly negative. Contrast-enhanced CT of the upper abdomen: In the arterial phase of enhanced liver parenchyma, patchy weak enhancement shadow was seen, and the enhancement degree was weaker than that of the rest of liver parenchyma. The three hepatic veins were slender, and the portal and hepatic veins were clear. The diameter of the portal vein was about 12.1 mm.liver parenchyma injury; Weak enhancement in liver parenchyma, considering the possibility of injury; Portal hypertension was suggested. The three hepatic veins were slender, Ascites. (Figures 3A,B). MRCP results showed no obvious abnormality. Color Doppler ultrasound of hepatic vein, portal vein and inferior vena cava showed portal vein widening and embolus band discharge (Figure 3C). Liver biopsy was not performed to onfirm the pathological diagnosis.

**FIGURE 3:**
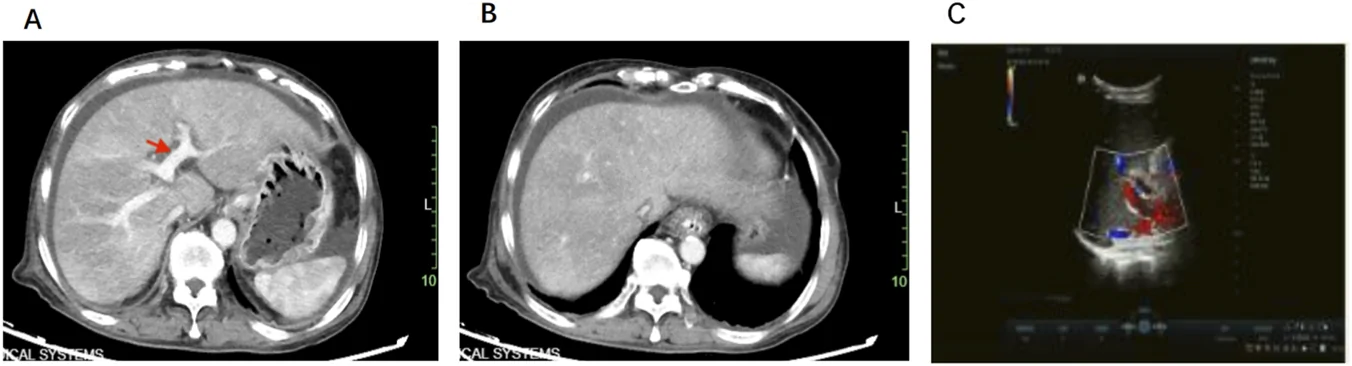
Multidisciplinary consultation and definitive diagnosis. **(A and B)** Contrast-enhanced CT of the upper abdomen (enous phase): Widening of the portal vein was seen, suggesting portal hypertension. The three hepatic veins were slender. Visible ascites (Pictures 13 and 18 of the venous phase in the attachment). **(C)** Color Doppler ultrasound revealed dilatation of the portal vein, and a thrombus needs to be ruled out.

#### 2.3.3 Differential diagnosis of HSOS

The differential diagnosis of HSOS is detailed in Table 3.

**TABLE 3:** Differential diagnosis of hepatic sinusoidal obstruction syndrome (HSOS).

Differential feature	HSOS	Ovarian cancer	Budd-Chiari syndrome	Portal vein thrombosis	Cirrhosis with portal hypertension	Abdominal tuberculosis	Peritoneal carcinomatosis	Right heart failure
Primary pathogenesis	Hepatic sinusoidal and small hepatic vein endothelial injury, leading to venular fibrosis and obstruction, sinusoidal congestion, and portal hypertension	Malignant epithelial tumor of ovary; ascites due to peritoneal seeding, lymphatic obstruction, or hypoalbuminemia	Occlusion of main hepatic veins or suprahepatic IVC, causing hepatic outflow obstruction and sinusoidal hypertension	Thrombotic occlusion of portal vein, leading to presinusoidal portal hypertension	Diffuse hepatic fibrosis and regenerative nodules, resulting in sinusoidal portal hypertension	*Mycobacterium tuberculosis* infection of peritoneum, causing inflammatory exudation and ascites	Metastatic malignant cells seeding peritoneum, producing exudative ascites	Impaired right ventricular filling or contraction, causing systemic venous congestion and hepatic congestion
Key etiology/Risk factors	Pyrrolizidine alkaloids (Gynura segetum), chemotherapy, HSCT, immunosuppressive drugs	Age >50, BRCA mutation, family history, nulliparity, endometriosis, obesity	Myeloproliferative disorders, antiphospholipid syndrome, pregnancy, oral contraceptives, tumor invasion	Cirrhosis, malignancy, myeloproliferative disorders, abdominal infection, trauma, surgery	Chronic hepatitis B/C, alcohol, NASH, autoimmune hepatitis, biliary diseases	Pulmonary TB history, immunosuppression, malnutrition, endemic regions	Gastrointestinal (gastric, colorectal), pancreatic, ovarian, or breast primary malignancies	Cor pulmonale, right-sided valvular disease, constrictive pericarditis, cardiomyopathy, pulmonary hypertension
Ascites characteristics	Transudate or modified transudate; SAAG usually >11 g/L; low protein; typically not bloody	Exudate; SAAG <11 g/L; often bloody; elevated CA-125; positive cytology in advanced cases	Transudate or modified transudate; SAAG >11 g/L; high protein content (>25 g/L)	Transudate; SAAG >11 g/L (pre-sinusoidal); variable protein content	Transudate; SAAG >11 g/L; low protein; may become exudative with SBP	Exudate; SAAG <11 g/L; lymphocyte-predominant; elevated ADA; low glucose	Exudate; SAAG <11 g/L; often bloody; positive cytology; elevated LDH and CEA	Transudate; SAAG >11 g/L; high protein (pericardial disease); low WBC count
Liver function tests	Elevated bilirubin (direct dominant); moderate elevation of AST/ALT; hypoalbuminemia; prolonged INR; elevated GGT/ALP in cholestatic pattern	Normal or mild LFT elevation; hypoalbuminemia from protein loss; elevated CA-125, CA19-9, CEA	Markedly elevated AST/ALT in acute phase; hyperbilirubinemia; elevated ALP/GGT; hypoalbuminemia in chronic phase	LFTs may be normal or mild elevation; bilirubin may be slightly elevated; liver synthetic function preserved unless underlying cirrhosis	Variable: elevated AST/ALT (AST>ALT), bilirubin, ALP/GGT; hypoalbuminemia; prolonged INR; thrombocytopenia	LFTs usually normal or mild elevation; hypoalbuminemia from chronic inflammation; elevated ESR/CRP	LFTs often normal; hypoalbuminemia; elevated tumor markers (CEA, CA19-9, CA-125)	Mild elevation of AST/ALT (congestive hepatitis); hyperbilirubinemia (mild); elevated GGT; elevated BNP/NT-proBNP
Key imaging findings	Patent hepatic veins; “map-like” or “mottled” liver enhancement; periportal edema; hepatomegaly; ascites; gallbladder wall thickening; IVC stenosis or compression	Ovarian mass with solid/cystic components; omental caking (“omental cake”); peritoneal nodules; ascites; enlarged lymph nodes	Hepatic vein/IVC filling defect or occlusion; caudate lobe hypertrophy; “flip-flop” enhancement pattern; hepatic vein-IVC pressure gradient	Portal vein filling defect on CT/MRI/ultrasound; cavernous transformation (chronic); collaterals (coronary vein, splenic vein); splenomegaly	Nodular liver surface; liver morphological changes (right lobe atrophy, left lobe hypertrophy); splenomegaly; portal vein dilation; varices	Thickened peritoneum (smooth or nodular); omental thickening; mesenteric lymphadenopathy (central necrosis); ascites; ileocecal thickening	Nodular peritoneal thickening; omental caking; ascites; primary tumor may be visible; multiple peritoneal implants	Dilated IVC and hepatic veins; hepatomegaly; ascites; pleural effusion; cardiac enlargement; pericardial thickening (constrictive pericarditis)
Key diagnostic procedures	Liver biopsy (pathognomonic: Venular fibrosis, centrilobular congestion, subendothelial edema); HVPG measurement; history of PA exposure	Pelvic ultrasound/CT/MRI; tumor markers (CA-125, HE4); ascites cytology; laparoscopy with biopsy; surgical staging	Doppler ultrasound; CT/MRI with hepatic venography; hepatic vein catheterization; HVPG measurement	Doppler ultrasound; CT angiography/MRV; portal venography; evaluation for underlying prothrombotic conditions	Liver elastography (FibroScan); liver biopsy; endoscopy for varices; etiology workup (HBV, HCV, alcohol, *etc.*)	Ascites ADA; ascites AFB smear/culture; T-SPOT.TB/PPD; peritoneal biopsy (laparoscopic); imaging findings	Ascites cytology; CT abdomen/pelvis with contrast; PET-CT; laparoscopy with peritoneal biopsy; tumor markers	Echocardiogram; ECG; BNP/NT-proBNP; chest X-ray; right heart catheterization; CT for pericardial calcification
Defining diagnostic features	History of pyrrolizidine alkaloid exposure; patent hepatic veins; characteristic liver biopsy (small hepatic vein obstruction/fibrosis); no evidence of cardiac or large-vessel disease	Adnexal mass on imaging; elevated CA-125/HE4; positive ascites cytology; omental caking; histologic confirmation	Demonstration of hepatic vein or IVC occlusion/stenosis on imaging; elevated hepatic vein pressure gradient; caudate lobe hypertrophy	Visualization of thrombus in portal vein on Doppler, CT, or MRI; cavernous transformation in chronic cases; portal hypertension signs	Stigmata of chronic liver disease; nodular liver and pseudolobules on biopsy; portal hypertension with varices/splenomegaly	Lymphocytic ascites with high ADA; positive T-SPOT.TB or PPD; peritoneal biopsy with caseating granulomas; response to anti-TB therapy	Positive peritoneal cytology or biopsy confirming malignant cells; omental/peritoneal nodules on imaging; known primary malignancy	Elevated JVP; peripheral edema; hepatomegaly with pulsations (TR); abnormal echocardiogram; elevated BNP/NT-proBNP; response to diuretics

Abbreviations: HSOS, hepatic sinusoidal obstruction syndrome; IVC, inferior vena cava; HSCT, hematopoietic stem cell transplantation; SAAG, serum-ascites albumin gradient; LFT, liver function test; AST, aspartate aminotransferase; ALT, alanine aminotransferase; ALP, alkaline phosphatase; GGT, gamma-glutamyl transferase; INR, international normalized ratio; HVPG, hepatic venous pressure gradient; NASH, nonalcoholic steatohepatitis; ADA, adenosine deaminase; LDH, lactate dehydrogenase; CEA, carcinoembryonic antigen; SBP, spontaneous bacterial peritonitis; BNP, B-type natriuretic peptide; PA, pyrrolizidine alkaloid; TB, tuberculosis; ESR, erythrocyte sedimentation rate; CRP, C-reactive protein; TR, tricuspid regurgitation; JVP, jugular venous pressure.

#### 2.4 Targeted treatment and clinical course

The patient was transferred to the Infectious Disease department for specialized management. During hospitalization in the Department of Infectious Diseases, the patient received a comprehensive treatment regimen as follows:

1. Anticoagulation therapy: Bemiparin sodium injection (3,500 IU, subcutaneous injection, qd) was administered for a duration of 3 weeks. No oral anticoagulant was prescribed thereafter.
2. Hepatoprotective agents: Compound ammonium glycyrrhizinate, polyene phosphatidylcholine injection, and ursodeoxycholic acid capsules were administered for liver protection and cholestasis management.
3. Albumin supplementation: Human albumin (10 g, intravenous drip, qd) was given for 2 weeks to correct hypoalbuminemia and maintain plasma oncotic pressure.
4. Diuretic therapy: Furosemide tablets (20 mg, bid) were prescribed to manage fluid overload and reduce ascites.
5. Abdominal paracentesis and drainage: Abdominal paracentesis was performed to drain ascitic fluid, thereby alleviating intra-abdominal pressure, relieving symptoms of abdominal distension, and preventing complications associated with massive ascites.

As a result, abdominal distension significantly improved, drainage volume decreased, and the patient was discharged on 2 September 2025.

#### 2.5 Follow-up and recurrence management

The patient was readmitted for follow-up on 24 September 2025. Metabolic status: laboratory tests revealed severely uncontrolled diabetes, with a glucose level of 35.23 mmol/L and an HbA1c of 10.7%. The ascites increased again. Liver function: recurrent ascites and fluctuating liver enzymes were observed, including elevated bilirubin and bile acids. Liver imaging was not performed during follow-up. Secondary Management: Paracentesis was repeated to relieve abdominal distension. Symptomatic treatments such as liver protection, anticoagulation and diuresis were continued. Bemiparin sodium injection (3,500 IU, subcutaneous injection, qd) was administered for a duration of 3 weeks. The patient’s condition stabilized before the second discharge.

## 3 Scientific concerns

The patient underwent laparoscopy and bilateral adnexectomy before completing liver specialist consultation, collecting the history of taking Chinese herbal medicine in detail, and completing the liver imaging examination, which was the oversight of the diagnosis and treatment process and the limitation of this case study.

The diagnosis is largely dependent on the history of exposure to herbs. RUCAM and other structured causality assessment tools for drug induced liver injury were not applied in this case. This is a limitation of this study.

## 4 Discussion

This case highlights a classic diagnostic dilemma where Hepatic Sinusoidal Obstruction Syndrome (HSOS) induced by Gynura segetum (Tusanqi) was misidentified as ovarian cancer (Lin et al., 2011). Clinically, HSOS presents with a rapid onset of abdominal distension, recalcitrant ascites, and hepatomegaly, symptoms that overlap significantly with the malignant ascites typically observed in advanced abdominal malignancies. In this patient, the striking elevation of serum tumor markers, including CA125 and HE4, alongside imaging findings suggesting adnexal abnormalities and peritoneal thickening, created a “clinical masquerade” that strongly pointed toward stage III or IV ovarian carcinoma with peritoneal carcinomatosis.

The primary cause of misdiagnosis in this case was the non-specific nature of early HSOS markers. Elevated CA125 is not exclusively indicative of malignancy; it can be significantly increased in response to peritoneal irritation caused by massive benign ascites or inflammatory liver disease (Topalak et al., 2002). The mechanism of CA125 elevation: ascites, peritoneal interstitial edema and portal hypertension caused peritoneal mesothelial cells to secrete CA125 and HE4, which explained the false positive of tumor markers. Pelvic magnetic resonance imaging showed increased soft tissue shadows in the bilateral adnexal region, combined with a large amount of fluid in the abdominal and pelvic cavity, which was similar to the imaging of ovarian cancer. Furthermore, the lack of clinical vigilance regarding plant-derived drug-induced liver injury (DILI) led to an incomplete initial investigation of the patient’s herbal medicine history (Halegoua-DeMarzio and Navarro, 2025). According to the Nanjing Criteria, the definitive diagnosis of HSOS relies on a confirmed history of PA ingestion and characteristic imaging features, such as the “geographic” or patchy enhancement pattern of the hepatic parenchyma and the narrowing of hepatic veins on contrast-enhanced CT or MRI (Zhang et al., 2025). It was only through subsequent detailed history-taking and re-evaluation of the MRCP and CT scans that the diagnosis was corrected. In the initial diagnosis, CT examination of the upper and lower abdomen was missed, and the problem of liver and portal vein was not found in time.

In conclusion, this case serves as a critical reminder for clinicians, particularly gynecologists and oncologists. When encountering female patients with unexplained ascites and elevated tumor markers, it is essential to look beyond the possibility of malignancy. A thorough inquiry into the use of traditional Chinese medicines or folk remedies, combined with specialized vascular imaging of the liver, is paramount to avoiding unnecessary invasive surgeries and ensuring the timely initiation of appropriate anticoagulation therapy (Wang et al., 2022). To the best of our knowledge, there are no previously published case reports or case series documenting PA-HSOS being misdiagnosed as ovarian malignancy or peritoneal carcinomatosis resulting in surgical intervention. The present case represents the first documented instance of this specific diagnostic pitfall. While CA125 elevation in PA-HSOS is a well-recognized phenomenon in the Chinese literature, and while other liver diseases have been reported to mimic ovarian malignancy, the intersection of these two—PA-HSOS masquerading as ovarian cancer leading to laparoscopy and bilateral salpingo-oophorectomy—has not been previously reported. This case fills this gap in the literature and has important cautions for clinical practice.

## Data Availability

The original contributions presented in the study are included in the article/supplementary material, further inquiries can be directed to the corresponding authors.
